# Beyond Discovering the Viral Agents of Acute Gastroenteritis

**DOI:** 10.3201/eid1908.130773

**Published:** 2013-08

**Authors:** Roger Glass

**Affiliations:** National Institutes of Health, Bethesda, Maryland, USA

**Keywords:** gastroenteritis, acute gastroenteritis, viral gastroenteritis, transmission, etiology, norovirus, rotavirus, astrovirus, enteric adenovirus, sapovirus, outbreak, diarrhea, viruses, enteric infections

In the field of enteric microbiology, every major advance in diagnostics has enhanced our understanding of the etiology of gastroenteritis, the role of each pathogen, the different modes of transmission, and control methods that should be considered. Before 1970, >80% of gastroenteritis episodes did not have an etiologic diagnosis; these cases were attributed to weaning, malnutrition, or, most often, idiopathic causes. Then, in 1972, electron microscopists began to examine fecal specimens from patients with acute gastroenteritis, and within a decade, a collection of novel enteric viruses had been discovered: Norwalk virus (noroviruses), rotaviruses, astroviruses, enteric adenoviruses, classic human caliciviruses (sapoviruses), and others. Together, these novel viruses explained most of the severe cases of diarrhea in children and most of the acute outbreaks of disease occurring in the industrialized world. 

For rotavirus, research progressed, and simple, sensitive, and inexpensive immunoassays soon displaced electron microscopy as the diagnostic test of choice available in laboratories around the world. On the basis of assay results, rotavirus was determined to be the most common cause of severe diarrhea in children worldwide. By contrast, the other enteric viruses, which are shed in lower quantities, could not be grown in culture and could not initially be detected by simple immunoassays. Consequently, for 2 decades, electron microscopy remained the main diagnostic tool available in a few research laboratories worldwide. 

A key breakthrough came around 1990 when the first genetic sequences of these novel viruses were decoded, opening the way for new molecular diagnostics and reverse transcription PCR (RT-PCR). Noroviruses appeared to be prevalent in young children and in adults, including the elderly, and were soon recognized as the most common cause of outbreaks of and hospitalizations for gastroenteritis in industrialized countries. 

What we have learned about rotaviruses and noroviruses alone has revolutionized our understanding of the critical role they play in public health and placed the other novel enteric viruses as second-tier agents. However, we are still in the discovery phase with these new viruses, learning about their public health impact worldwide and developing methods for their control. For rotaviruses, the mode of transmission is still in question, but widely introduced vaccines have provided a highly effective measure of disease control. For noroviruses, outbreaks can often be traced to fecally contaminated food or water and person-to-person transmission. The same diagnostics that have enabled our understanding of the high prevalence of disease worldwide have also enabled genetic fingerprinting of strains, a process critical to seeking a common source of exposure and to tracing routes of transmission back to fecally contaminated food, water, or food handlers. Noroviruses were first associated with outbreaks of disease; however, recognition of their key role in the hospitalization of adults with diarrhea indicates that those affected have incomplete immunity against norovirus or that strain diversity is too great to provide adequate cross-protection in persons who have been previously infected with other strains. Noroviruses also demonstrate a unique genetic susceptibility related to secretor histo-blood group antigens, so not all humans are equally susceptible to norovirus infection.

Limitations in our current knowledge of noroviruses and the other novel enteric viruses, excluding rotavirus, can be addressed by research over the next decade. RT-PCR opened the door to detecting these pathogens in research studies, but routine knowledge of the disease they cause remains limited by the lack of widely used, inexpensive, and simple diagnostics tests in the field or at the bedside. This lack of routine tests prevents health care providers from making an etiologic diagnosis and limits our understanding of transmission within hospitals and among the staff and in the community. In developing countries, these pathogens often occur together as mixed infections, making it difficult to assess whether the virus is really contributing to the disease process or merely passing through without replicating or inducing an immune response. RT-PCR and quantitative PCR can detect even a few viral particles, and shedding of these viruses in small numbers can continue for weeks or months, making it difficult to distinguish patients with real disease caused by the virus from those who have long since recovered from their infections but are still shedding virus.

This issue of Emerging Infectious Diseases provides readers with an opportunity to become informed about the latest updates in our knowledge of the viral agents of gastroenteritis, the global impact of the disease they cause, and prospects for their treatment and prevention. There is much new today, from the genetic human leukocyte antigen–related predisposition of disease to advances in vaccine development, understanding the diversity and evolution of strains, and testing of new solutions for environmental control, and there is also a robust research agenda ahead. Despite all the attention society has placed on ensuring access to clean food and water, sewage control, and handwashing, gastrointestinal illnesses remain one of the most common afflictions of humankind. We still have a lot to learn.

